# Structural Basis of Botulinum Toxin Type F Binding to Glycosylated Human SV2A: *In Silico* Studies at the Periphery of a Lipid Raft

**DOI:** 10.3390/biom12121821

**Published:** 2022-12-06

**Authors:** Fodil Azzaz, Didier Hilaire, Jacques Fantini

**Affiliations:** 1 Fodil Azzaz, INSERM U_1072, Faculté de Médecine Nord, Bd Pierre Dramard, University of Aix-Marseille, 13015 Marseille, France; 2 DGA (Direction Générale de L’armement)—DGA Maîtrise NRBC, 91710 Vert le Petit, France

**Keywords:** molecular modeling, gangliosides, botulinum neurotoxin, SV2, receptor, structural biology, molecular dynamics simulation, lipid raft

## Abstract

Botulinum neurotoxins are the deadliest microbial neurotoxins in humans, with a lethal dose of 1 ng/kg. Incidentally, these neurotoxins are also widely used for medical and cosmetic purposes. However, little is known about the molecular mechanisms that control binding of botulinum neurotoxin type F1 (BoNT/F1) to its membrane receptor, glycosylated human synaptic vesicle glycoprotein A (hSV2Ag). To elucidate these mechanisms, we performed a molecular dynamics simulation (MDS) study of initial binding kinetics of BoNT/F1 to SV2A. Since this toxin also interacts with gangliosides, the simulations were performed at the periphery of a lipid raft in the presence of both SV2A and gangliosides. Our study suggested that interaction of BoNT/F1 with SV2A is exclusively mediated by N-glycan moiety of SV2A, which interacts with aromatic residues Y898, Y910, F946, Y1059 and H1273 of this toxin. Thus, in contrast with botulinum neurotoxin A1 (BoNT/A1), BoNT/F1 does not interact with protein content of SV2A. We attributed this incapability to a barrage effect exerted by neurotoxin residues Y1132, Q1133 and K1134, which prevent formation of long-lasting intermolecular hydrogen bonds. We also provided structural elements that suggest that BoNT/F1 uses the strategy of BoNT/A1 combined with the strategy of botulinum neurotoxin type E to bind N-glycan of its glycoprotein receptor. Overall, our study opened a gate for design of a universal inhibitor aimed at disrupting N-glycan–toxin interactions and for bioengineering of a BoNT/F1 protein that may be able to bind protein content of synaptic vesicle glycoprotein for therapeutic purposes.

## 1. Introduction

Botulinum neurotoxin type F1 (BoNT/F1) is a potent agent, produced by *Clostridium botulinum*, that infects the nervous system of humans and causes botulism [1]. This disease occurs after cleavage of SNARE proteins (comprising syntaxin 1, VAMP1-3 and SNAPE-25), which are responsible for neurotransmitter release and can eventually lead to death via respiratory failure [2]. Typically, botulinum neurotoxins are classified into eight serotypes (from A to G and X), among which serotypes A, B, E and F were reported to be associated with human botulism [3,4]. The A serotype (BoNT/A) is the most potent in humans, followed by B, E and F [5].

**Figure 1:**
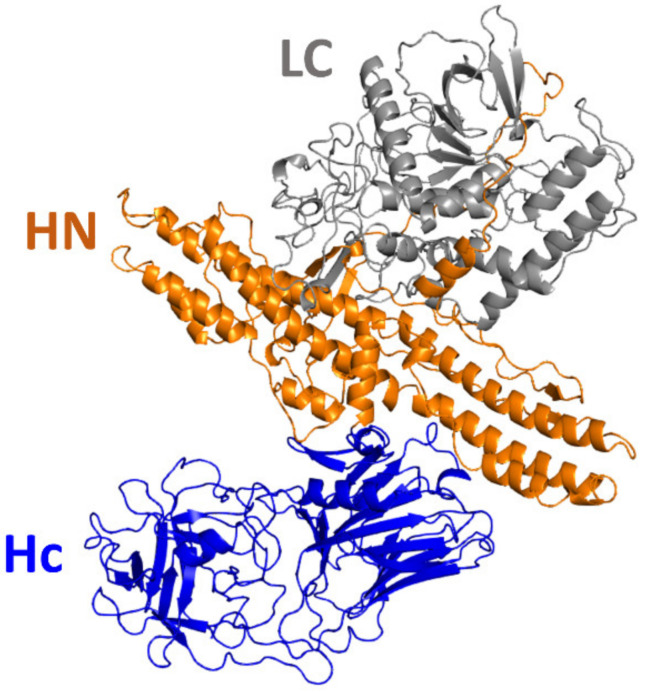
Molecular structure of the full length of BoNT/A1, experimentally resolved with X-ray diffraction. Botulinum neurotoxins are each composed of an LC domain, an HN sub-domain and an Hc sub-domain. The Hc sub-domain is responsible for binding of neurotoxin to neuronal membranes. Once attached, toxin is endocytosed by the cell. Then, upon acidification of the inside of the vesicle, the HN sub-domain is responsible for translocation of the LC domain from the vesicle to the cytoplasm. Finally, the LC chain of neurotoxin cleaves the SNARE proteins, which prevents fusion of the neurotransmitter vesicles with the plasma membrane, resulting in inhibition of neurotransmission. Hc is depicted in blue, HN is depicted in orange and LC is depicted in white.

Regardless of the serotype, botulinum neurotoxins (Figure 1) are structurally defined by a light chain (LC, a metalloprotease that is responsible for cleavage of SNARE proteins) attached to a heavy chain (HC) via a set of non-covalent interactions and a disulfide bridge [6]. The HC domain is further divided into two translocation subdomains, respectively referred to as HN (responsible for translocation of the LC into cytoplasm of neural cells) and Hc (the C-terminal subdomain of HC). There are up to nine different subtypes of BoNT/F, with sequence differences ranging from 4 to 30%; most of the differences are in the light-chain-coding region.

As in the case of BoNT/A1, BoNT/F1 Hc binds to neurons with a dual-interaction process that involves (i) recognition of synaptic vesicle glycoproteins A, B and C (SV2A, SV2B and SV2C) and (ii) binding to gangliosides, especially GT1b and GD1a, which display the highest affinity for this toxin. The latter interaction involves a ganglioside binding site (GBS) conserved in serotypes A, B, E, F and G as well as in tetanus neurotoxin [7,8]. BoNT/A1 inhibits binding of BoNT/F1 to its receptor, suggesting that both toxins share the same binding area.

Potential danger of these neurotoxins to human health notwithstanding, BoNTs are clinically exploited for medical purposes. Although BoNT/A1 remains the most-used toxin due to its highest potency in humans, clinical application of BoNT/F1 is growing in interest for patients that have developed antibodies directed against BoNT/A1, making treatment with BoNT/A1 significantly less effective [9]. In addition, BoNTs can be easily derived as biological weapons and are classified by the Biosafety Institute as a serious threat [10].

Despite the clinical interest in BoNT/F1, the molecular mechanisms that control interaction of this toxin with the SV2A receptor are poorly understood. This lack of knowledge is a handicap to designing bioengineered toxins with improved therapeutic properties, as well as antagonist drugs in the event of intoxication. To provide this knowledge, we performed a molecular dynamics simulation of BoNT/F1 in complex with its membrane receptor, glycosylated human SV2A (hSV2Ag), at the periphery of a lipid raft.

## 2. Materials and Methods

### 2.1. Molecular Modeling and Systems Set Up

*Building of toxin models:* The Hc sub-domain of BoNT/A1 is from the PDB file 5JLV [11] while the Hc-subdomain of BoNT/F1 is from the crystal structure 3FUQ [8]. The missing region was built with SWISS-MODEL (https://swissmodel.expasy.org/; accessed on 1 November 2022).

*Building of hSV2Ag models:* The full length of the hSV2A receptor was built via ab initio modeling in Robetta (https://robetta.bakerlab.org/; accessed on 1 November 2022), using the RosseTTAFold method. This modeling method was selected for its accuracy in respecting topology of membrane proteins [12,13]. Amino acids 1 to 161, which represent the intracellular domain of hSV2A, were removed due to their intrinsic disorder. Then, hSV2A was submitted to energy minimization with the Polak-Ribière algorithm of HyperChem, using the CHARMM force field (http://www.hypercubeusa.com/; accessed on 1 November 2022). Finally, N-glycosylation of hSV2A at position N573 was built using the tool “PDB reader” on CHARMM-GUI [14].

*Docking of the neurotoxins to hSV2Ag:* BoNT/A1 and BoNT/F1 were docked near the surface of hSV2A that is homologous to the binding site of BoNT/A with SV2C. Then, each complex was minimized to improve the intermolecular contacts between (i) toxin and N-glycan, (ii) toxin and protein content of hSV2Ag and (iii) N-glycan and protein content of hSV2Ag. Both toxins were docked in the same position and orientation to compare their mechanisms of interaction with hSV2A.

*Building of the lipid raft and the lipid bilayer:* We built a lipid raft composed of ganglioside GT1b and cholesterol. The initial coordinates and CHARMM topology of cholesterol and ganglioside molecules that constitute the GT1b/cholesterol cluster were obtained with CHARMM-GUI, using the tools “membrane builder” and “glycolipid modeler” [15,16]. Each element was manually assembled in VMD software to build a lipid raft that comprised 36 molecules of GT1b and 36 molecules of cholesterol. The bilayer membrane, consisting of POPC and cholesterol (CLR) at a ratio of 1:1, was built using the tool “Membrane Builder” on CHARMM-GUI [15].

*Assembly of each element for system setup:* First, the lipid raft was merged with the membrane bilayer using VMD software (version 1.9.4 for Windows) to obtain a lipid raft that was inserted into the POPC:CLR membrane. All POPC and cholesterol molecules that overlapped the molecular surface of the lipid raft were removed. Next, the BoNT/A1–hSV2Ag complex (or BoNT/F1–hSV2Ag) was inserted at the periphery of the lipid raft. POPC, the gangliosides and the cholesterol molecules that overlapped the molecular surface of the membrane protein were removed. Then, the systems were solubilized with a TIP3P water model and neutralized with Na^+^ and Cl^−^ ions at a final concentration of 0.15 mol/L, using the tools “Add Solvation Box” and “Add Ions” of VMD software.

### 2.2. Molecular Dynamics Simulation

Molecular dynamics simulations were performed as described in a previous work [17]. Briefly, the systems were simulated using NAMD 2.14 for Windows 10, coupled with the CHARMM36m force field [18,19]. The apolar domains of the systems were melted for 1 ns in an NVT ensemble. Thereafter, equilibration of the systems with constraints on protein was performed at a constant temperature (310 K) and constant pressure (1 atm) for 10 ns, followed by 10 additional ns with constraint on the backbone. The non-constrained runs were performed for 100 ns, with a time step of 2 fs. The coordinates during production runs were backed up every 0.1 ns, corresponding to 50,000 steps. The cutoff for calculation of non-covalent interaction was set at 12 Å, and the PME algorithm was used for calculation of long-range electrostatic interactions in a periodic system.

### 2.3. Analysis

All snapshots presented in this study were taken using the VMD rendering mode and PyMoL. The root mean square deviation of BoNT/A1 and BoNT/F1 were measured using the plugin “RMSD trajectory tool” on VMD. The hydrogen bonds were calculated between 20 and 100 ns, with a cutoff of 3 Å and an angle cutoff of 20° between partners (donor and acceptor).

### 2.4. Molecular Modeling of BoNT/F Subtypes

The sequences of all subtypes of BoNT/F from F2 to F9 were retrieved on the BoNTbase database (https://bontbase.org/; accessed on 1 November 2022), which stores the sequence of each known type and subtype of botulinum neurotoxin. Sequence coding for the Hc domain of each BoNT/F subtype was loaded on SWISS-MODEL in order to build models with homology. For each model, the crystal structure PDB: 3FUQ, containing the coordinates of the Hc domain of BoNT/F1, was used as a template.

## 3. Results

### 3.1. Protein–Protein Contacts between the Complexes BoNT/F1–hSV2Ag and BoNT/A1–hSV2Ag

Before starting any analysis, we made sure that the proteins built in our systems underwent no unrealistic conformational changes that could have been generated by unstable systems. To check this, we measured root mean square deviation (RMSD) of the toxins over time. The plots revealed that BoNT/F1 and BoNT/A1 had an average RMSD value around 2 Å, demonstrating stability of these structures (Figure S1). In addition, since hSV2Ag was obtained via ab initio modelling, we measured root mean square fluctuation (RMSF) and RMSD of hSV2Ag in the BoNT/A1 (Figure S2) and BoNT/F1 (Figure S3) systems. In both cases, the results showed that RMSF was equal or below 2 Å for the transmembrane domain (TMD) and the structured part of the luminal domain (LD), whereas RMSF was above 2 Å, reaching the maximum value of 7 Å for non-structured regions. RMSD of the TMD of hSV2Ag was transient at a value of 1.4 Å in the BoNT/A1 system (Figure S2) and 1.8 Å in the BoNT/F1 system (Figure S3). As for the neurotoxins, the RMSD and RMSF values suggest that hSV2Ag undergoes no artefactual conformational changes in either system.

**Figure 2:**
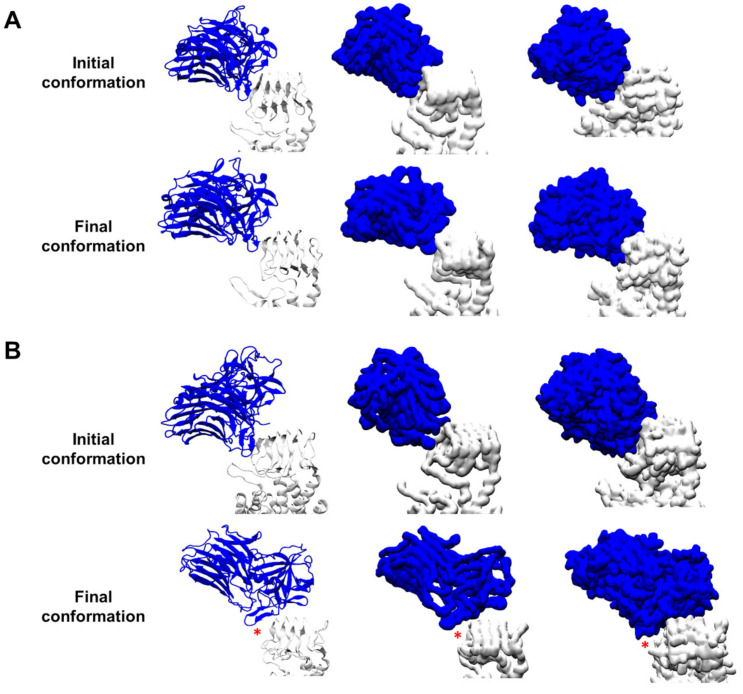
Evolution of the BoNT/A1–hSV2Ag and BoNT/F1–hSV2Ag complexes via MDS. Snapshots showing the initial and final conformations of the BoNT/A1–hSV2Ag complex (**A**) and the initial and final conformations of the BoNT/F1–hSV2Ag complex (**B**). BoNT/A1 and F1 are depicted as the color blue while hSV2Ag is represented as the color white. The left column shows an illustrated representation of the complexes. The middle column shows surface representation of the complexes that considers only the atoms of the backbone. The right column shows a surface representation of the complexes that considers all atoms (backbone and side chains). Red asterisks highlight a gap showing that there is no intermolecular contact between the toxin and the protein part of hSV2Ag.

**Figure 3:**
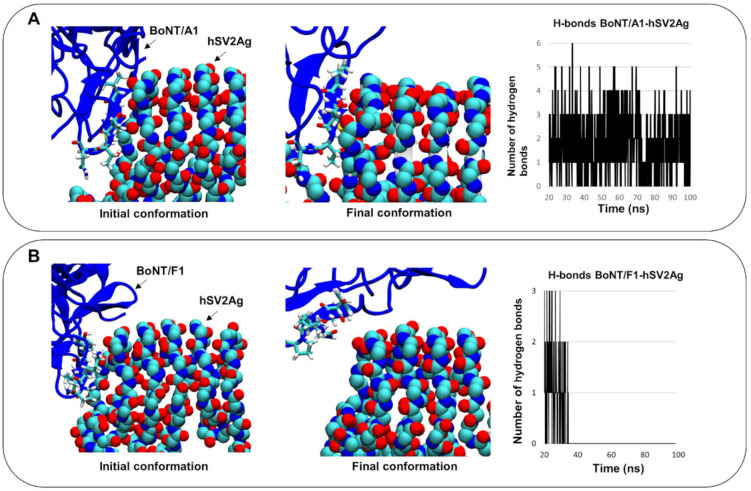
Molecular details of the interactions between BoNT/A1 or BoNT/F1 and hSV2Ag. Snapshots showing the molecular details of the initial and final conformations obtained for the BoNT/A1 complex and a plot presenting the number of hydrogen bonds formed by the complex (**A**). Similar data for the BoNT/F1–hSV2Ag complex (**B**). BoNT/A1 and BoNT/F1 are depicted as the color blue, and 1141-GSVMTT-1146 in BoNT/A1 and 1130-GVYQKP-1135 in BoNT/F1 are represented as lines colored according to atom name. The backbone of the membrane receptor hSV2Ag is depicted as spheres colored according to atom name (cyan for carbon, blue for nitrogen and red for oxygen)..

Then, we compared evolution of interaction between BoNT/F1 or BoNT/A1 with protein content of their membrane receptor, hSV2Ag. For this purpose, we placed neurotoxin in the same orientation and same position next to hSV2Ag and simulated each complex for 100 ns. The results in Figure 2 show snapshots of the initial and final conformations of both complexes. Even after 100 ns, BoNT/A1 still interacted with hSV2A (Figure 2A), which was not the case for BoNT/F1, as shown by a red asterisk that highlights a gap, indicating that there were no intermolecular contacts between both partners (Figure 2B). The molecular details of the intermolecular interaction are given in Figure 3. Remarkably, BoNT/A1 adjusted its beta-sheet structure, defined by the sequence 1141-GSVMTT-1146, along with the beta structure of hSV2Ag, defined by the sequence 574-STLFH-578 (Figure 3A). To go further in our analysis, we have plotted the number of hydrogen bonds formed by BoNT/A1 or BoNT/F1, with hSV2Ag between 20 and 100 ns. As shown in Figure 3A, both beta structures are maintained in interaction together via hydrogen bonds. In contrast, for BoNT/F1, the molecular details clearly show that beta structure 1130-GVYQKP-1135 fails to bind hSV2Ag. In fact, analysis of the plot reveals that hydrogen bonds were formed transiently in the early stages of the simulation (between 20 and approximately 35 ns), but these hydrogen bonds did not last in the middle or late stage of the simulation (Figure 3B).

Thus, our simulation predicted an absence of binding between BoNT/F1 and the protein part of hSV2Ag, suggesting that the beta sheet structure of BoNT/F1 is not compatible for interacting with its protein receptor.

### 3.2. Structural Comparison between BoNT/F1 and the Part of BoNT/A1 That Binds to Protein Moiety of hSV2Ag

**Figure 4:**
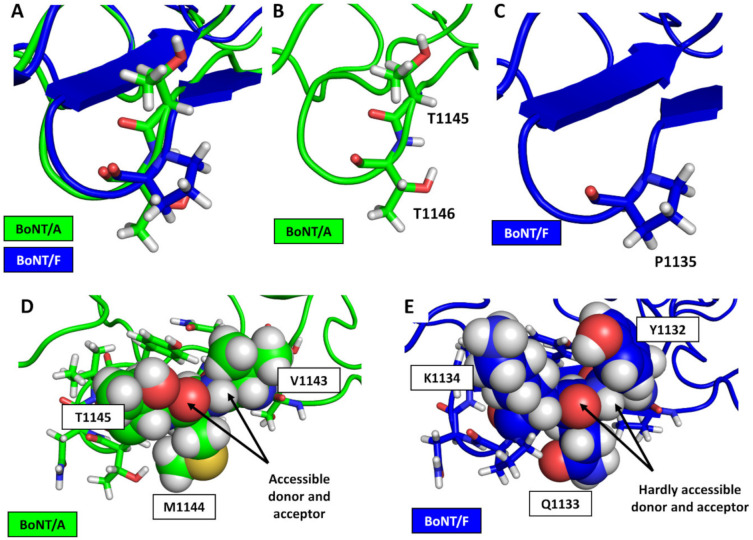
Structural analysis of BoNT/A and BoNT/F at the molecular level. Carbon alpha alignments of BoNT/A1 (green) and BoNT/F1 (blue), with T1145-T1146 of BoNT/A1 and P1135 of BoNT/F represented as lines (**A**). Zoomed-in images of residues T1145 and T1146 of BoNT/A1 (**B**) and residue P1135 of BoNT/F1 (**C**). Spherical representation of amino acids V1143 to T1145 of BoNT/A1 (**D**) and Y1132 to K1134 of BoNT/F1 (**E**).

We compared the crystallographic structure of BoNT/F1 (1278 amino acids) with that of BoNT/A1 to find clues that explain why BoNT/F1 does not bind protein content of its protein receptor. Based on our simulations, we propose three reasons. The first reason is the presence of proline, at position 1135 in the sequence of BoNT/F1, that occupies the same position as residue T1146 in the structure of BoNT/A. However, T1146 is a critical residue that mediates interaction with the protein receptor through formation of hydrogen bonds with the backbone of SV2 (Figure 4A–C) [20]. In contrast, proline is a residue that is unable to form hydrogen bonds via its lateral chain or its backbone. Thus, it represents the first constraint of BoNT/F1 binding to the beta structure of SV2.

The two other reasons concern the sequences V1131-T1141 and S1142-S1152 of BoNT/F and BoNT/A, respectively. The most striking difference is the presence of the residues Y1132 and K1134 in BoNT/F, which is structurally homologous to residues V1143 and T1145 in BoNT/A. The side chains of residues Y1132 and K1134 are longer and occupy a larger electronic space than the side chains of residues V1143 and T1145 occupy. Those differences have two impacts on binding with SV2. As shown in Figure 4D,E, compared to BoNT/A, electronic density of the R’R-N-H and R’R-C=O groups of the backbone of BoNT/F are shielded by electronic density and at the same time interact with the side chains of Y1132 and K1134. This complicates formation of long-term intermolecular backbone–backbone hydrogen bonds. In addition to a longer chain, Y1132 and K1134 had a wider range of possible torsions than residues V1143 and T1145 in BoNT/A1. This had the consequence of favoring intramolecular interactions with F1138, which was located on the opposite side of the beta sheet. Moreover, it is interesting to note that both effects were still operative in our simulation (Figure S4). Indeed, we could observe that Y1132 disrupts backbone–backbone hydrogen bonds that initially linked BoNT/F1 and SV2A (indicated by black asterisks in Figure S4A). In our simulation, residue Q1133 also appeared to form intramolecular hydrogen bonds with the backbone of BoNT/F1, indicated by a red asterisk in Figure S4A–C. This contributes to further decreasing possibility for intermolecular hydrogen bonding; it is not possible for its homologue M1144 in BoNT/A1 to form hydrogen bonds with either SV2A or BoNT/A1.

Thus, our computational data suggest that BoNT/F1 cannot bind protein content of SV2 because of the presence of proline that prevents formation of hydrogen bonds with its receptor, coupled with the presence of Y1132, Q1133 and K1134, which enhance stability of the beta-sheet structure of BoNT/F1 via privileging intramolecular interactions at the cost of intermolecular interactions with SV2.

### 3.3. Surface-Exposed Aromatic Amino Acids of BoNT/F1 Are Key Residues for Binding to N-Glycan of SV2

**Figure 5:**
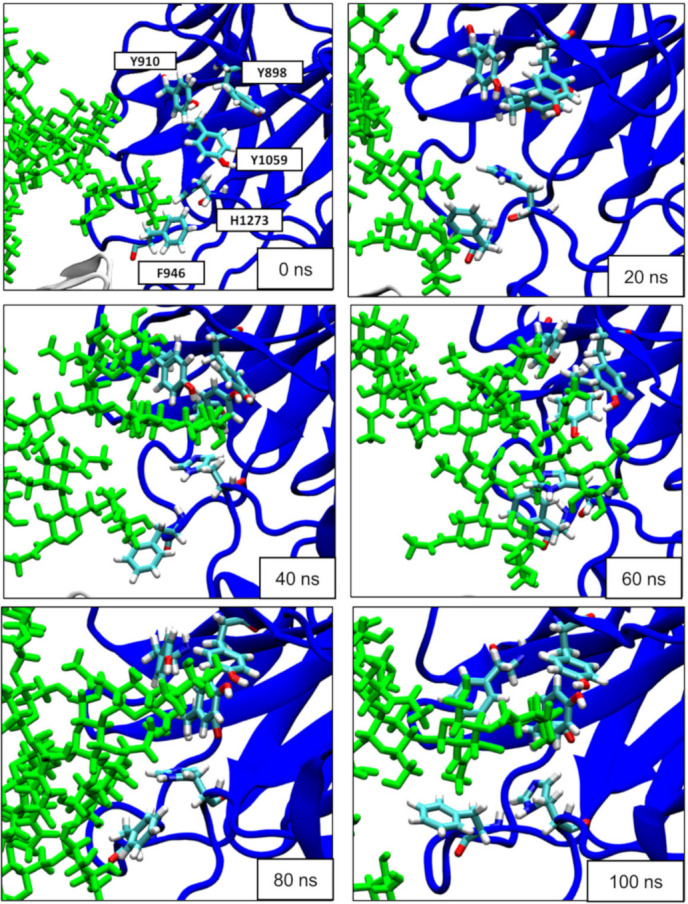
Evolution of interactions between BoNT/F1 and glycan content of hSV2Ag at 0, 20, 40, 60, 80 and 100 ns. BoNT/F1 is depicted as blue cartoon, the aromatic residues of BoNT/F1 that interact with the glycan are depicted as sticks colored according to atom name, and N-glycan is depicted as sticks colored in green.

Experimental data showed that BoNT/F1 binds its receptor SV2A only upon the presence of N-glycan [7,8]. Here, we also considered N-glycan at position 573 of SV2A to study how the sugar residues interacted with BoNT/F1. This N-glycan was selected because it is directly homologous to N-glycan attached at the position N559 of SV2C, which is known to bind to the BoNT/A1 surface [21]. Moreover, botulinum neurotoxin E (BoNT/E) (which, like BoNT/F1, fails to bind to the non-glycosylated) needs glycosylation at position 573 to bind SV2A [14]. BoNT/A also blocks binding of BoNT/E1 to SV2A [7], highlighting the importance of this N-glycan in mediation of binding of the toxins. Snapshots were taken at 0, 20, 40, 60, 80 and 100 ns to follow evolution of binding between BoNT/F and glycan content of SV2A. The visualization of the trajectory shows that the major points of contact between glycan and neurotoxin were mediated by aromatic residues Y898, Y910, F946, Y1059 and H1273. F946 was the first residue to interact with N-glycosylation; only after 40 ns did the sugar residues of N-glycan start to interact with residues Y898, Y910, Y1059 and H1273, forming a clamp around the sugar residue (Figure 5).

Thus, our data suggests that interaction of N-glycan with the surface of BoNT/F1 is essentially driven and maintained by aromatic residues.

### 3.4. Molecular Details Describing Interaction of BoNT/F1 with Lipid-Raft-Associated GT1b

**Figure 6:**
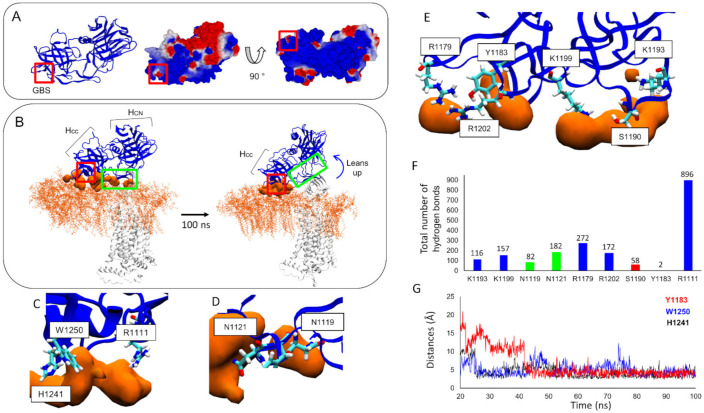
Electronic surface of the Hc domain of BoNT/F1, seen from the GBS side (middle) or the membrane surface side (right). The red color represents a globally anionic surface, the white color represents a neutral surface and the blue color represents a cationic surface (**A**). Neural-membrane-surface-induced conformational changes of the Hc domain of BoNT/F1. GT1b ganglioside molecules are represented as orange lines, and the GT1b atoms that were between 2.5 and 5 Å of the surface of BoNT/F1 are depicted as orange surfaces. BoNT/F1 is depicted as blue and hSV2Ag as white. Glycan content of hSV2Ag is not represented in these snapshots (**B**). Molecular details of residues in GBS interacting with gangliosides (**C**). Molecular details of interaction of two residues belonging to a flexible loop structure (**D**). Description of molecular interaction of residues that belong to a region in BoNT/F1 that is homologous to the synaptotagmin binding pocket (**E**). Histogram showing the total hydrogen bonds formed by each residue during the simulation (**F**). Plot showing the distance between gangliosides and aromatic-residue atoms that were engaged in CH-π or OH-π interactions (**G**).

Gangliosides are known to interact with BoNT/F1 and various other types of botulinum neurotoxin through recognition of the GBS [7]. In our simulation, the surface of the Hc domain of BoNT/F1 that was exposed on the surface of the membrane was globally cationic (represented as a blue surface) (Figure 6A). Thus, it was well suited for interacting with the anionic surface of GT1b gangliosides due to their negatively charged sialic acids. We described evolution of global orientation of the heavy chain on the surfaces of gangliosides (the gangliosides are depicted as orange lines, and the orange surface represents the gangliosides that were between 2.5 and 5 Å away from the neurotoxin surface). At the initial time, the Hc of BoNT/F1 was positioned vertically near the gangliosides (Figure 6B, left snapshot). During the simulation, we saw that neurotoxin progressively leaned on the edge of the Hcc domain (the C-terminal part of the Hc domain where the GBS is located, boxed in red) so that in the end, only the terminal part of the Hcc side remained in contact with the GT1b molecules, while the initial interaction with the back of the Hcc was disrupted (boxed in green) (Figure 6B, right snapshot). Molecular details of interaction of BoNT/F1 with the gangliosides are given. In the GBS region, BoNT/F1 chiefly interacted with gangliosides via its aromatic residues H1241 and W1250 (Figure 6C). Outside the GBS region, we found that amino acids N1121, N1119, R1179, R1202, K1199, S1190, K1193 and Y1183 also interacted with gangliosides essentially via hydrogen bonds (Figure 6D,E). To evaluate contribution of each amino acid over time, we plotted the total number of hydrogen bonds formed by each amino acid (Figure 6F). These results revealed that among all polar residues capable of interacting with gangliosides via hydrogen bonds and/or electrostatic interactions, the contribution of residue R1111 to bind GT1b is much stronger than that of all other residues, with nearly 896 hydrogen bonds formed. R1179 was the residue that formed the second most hydrogen bonds with GT1b: a total of 272. Then N1121, closely followed by R1202 and K1199, contributed almost equally, with a total of 182, 172 and 157 hydrogen bonds formed, respectively. Finally, K1193, N1119 and S1190 contributed moderately to the interaction with gangliosides, while Y1183 bound no GT1b via hydrogen bonds, with a total of only two hydrogen bonds formed. Aromatic residues typically interact with gangliosides via CH-π [22] or OH-π interactions [23]. We plotted the distance between the aromatic ring of each residue and the ganglioside atoms that were found to interact with Y1183, H1241 and W1250. Typically, for these interactions, we estimated that CH-π and OH-π interactions would be formed and maintained between 2.5 and 5 Å [23]. The interactions with gangliosides were first established with H1241 and W1250 at 25 ns and then remained stable throughout the simulation (Figure 6G). In the case of Y1183, interaction with gangliosides started at 40 ns and was maintained until the end of the trajectory.

Taken together, our computational data suggest that the Hcc domain of BoNT/F1 interacts with gangliosides via its aromatic residues Y1183, H1241 and W1250 through formation of CH-π and OH-π bonds. Cationic residues R1111, R1179, K1193, K1199 and R1202 were also critical to ganglioside binding, with R1111 displaying the highest contribution. Finally, we identified a set of polar residues involved in the binding, i.e., N1119, N1121 and S1190, among which N1121 showed the strongest contribution while S1190 showed the weakest.

## 4. Discussion

*In silico* approaches provide interesting mechanistic insights into protein–membrane interactions at the atomic scale [24,25]. Recently, we used MDS to study evolution of BoNT/A in complex with hSV2Ag, glycosylated human SV2C at position N559 and doubly glycosylated human SV2C at position N480 and N559 [17]. Lack of structural details of BoNT/F1interaction with membrane receptors made understanding how this neurotoxin invades neural cells a tough challenge. The scope of the present study was to compare protein–protein interaction of BoNT/F1 with hSV2Ag vs. that of BoNT/A1 with hSV2Ag and to describe the molecular mechanisms through which BoNT/F1 interacts with its membrane receptors. To the best of our knowledge, this is the first time MDS was used to study the conformational landscape of BoNT/F1 bound to its membrane receptor in a neural membrane context.

Our data suggest that BoNT/A1 can bind to protein content of hSV2Ag, which is consistent with in vitro pull-down assays showing that BoNT/A1 binds to non-glycosylated SV2A [7,26]. In the case of BoNT/F1, published experimental studies support the idea that BoNT/F1 fails to bind to non-glycosylated SV2, so BoNT/F1 requires the presence of glycosylation to bind to its receptor [7,8]. These experimental data have two possible interpretations: (i) neurotoxin binds partially to protein content of the receptor, and the presence of glycosylation is required to stabilize that binding; or (ii) neurotoxin does not recognize protein content of the receptor at all. Through prediction of the absence of long-lasting and stable contacts between the two protein partners, our MDS converged with experimental data. In addition, our in-depth structural analysis provided valuable clues as to why BoNT/F1 cannot bind to protein content of SV2. Nowadays, BoNT/F1 has interesting medical applications, as it can be administered to patients that have developed antibodies directed against BoNT/A1, which is the most clinically used botulinum neurotoxin [27,28]. In this respect, our data could be exploited to design bioengineered BoNT/F with enhanced therapeutic efficacy, as has been carried out with BoNT/B to increase its potency in humans [29,30,31].

**Figure 7:**
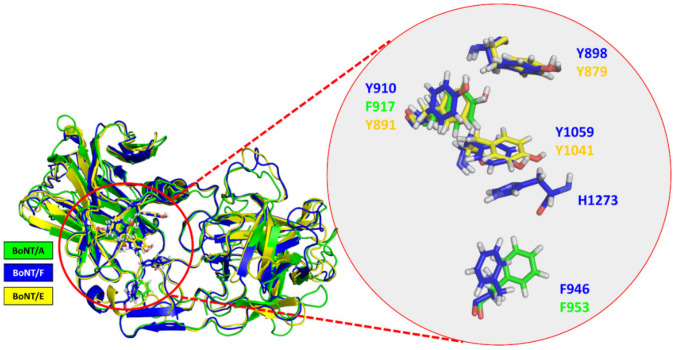
Structural alignment of BoNT/A1 and BoNT/E1 with the structure of BoNT/F1 and focus on the aromatic residues that interact with glycan. BoNT/A1 is depicted as the color green; BoNT/F1 and BoNT/E1 are colored in blue and yellow, respectively.

**Figure 8:**
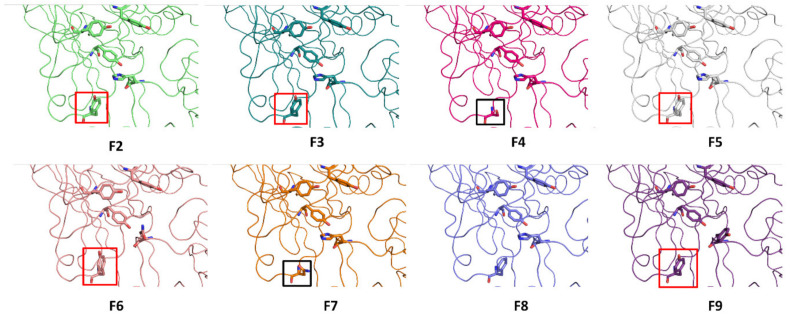
Structure of all subtypes of BoNT/F obtained via homology modeling, using crystal structure PDB: 3FUQ as a template.

Thereafter, we explored evolution of binding of BoNT/F1 to glycan content of hSV2Ag. No experimentally acquired structural data describing the interactions of the glycan–BoNT/F complex are currently available, probably due to difficulty of obtaining a periodic crystal, since BoNT/F1 does not bind directly to protein content of SV2. Our computational data suggests that N-glycan interacts with aromatic residues Y898, Y910, F946, Y1059 and H1273. BoNT/A1 and BoNT/E1 are toxins that also bind to glycan of SV2A [14]. To assess if there is a link between the contact points proposed by our MDS and those of the other toxins, we performed a structural alignment of BoNT/A1 and BoNT/E1 with BoNT/F1. F946 appeared to be structurally homologous to F953 of BoNT/A1 and Y910, and Y1059 appeared homologous to residues Y879 and Y1041 in BoNT/E1 (Figure 7). We also found that the amino acids that are key to interaction with glycan (Y898, Y910 and Y1059 in BoNT/F1) were well conserved among all BoNT/F subtypes and in BoNT/E1. On the other hand, aromatic residue F946, which was structurally homologous to F953 in BoNT/A1, was conserved in subtype F8 and replaced by a tyrosine that probably fills the role of phenylalanine (Figure 8) in subtypes F2, F3, F5, F6 and F9 (boxed in orange). In contrast, this phenylalanine was not preserved in subtypes F4 and F7, which have instead serine in F4 and arginine in F7 (boxed in black) (Figure 8). The residues of BoNT/A1 and BoNT/E1 mentioned in Figure 7 were experimentally validated with mutagenesis studies. Indeed, BoNT/E1 showed a significant decrease in its binding to SV2 and inhibition of its neurotoxicity when Y879 or Y1041 were mutated with alanine [32]. In the case of BoNT/A1, the residue F953 was identified to be a major contact point for binding of neurotoxin with its membrane receptor [11]. To think that BoNT/F1 conserved the major contact points of BoNT/A1 and BoNT/E1 to bind glycan is rational, and preservation of the residue F953 could be one of the reasons why the Hc domain of BoNT/F1 interferes with uptake of BoNT/E1 via increasing its paralytic half-time [7]. F910 is homologous to F917: a residue of BoNT/A1 we found interacting with N-glycan of hSV2A and hSV2C in MDS [17]. It is reasonable to assume that residues F910 and H1273 participate in binding of glycan due to their close vicinity with the major points of contact (Figure 7). Based on our theoretical explanation of the binding mechanism of BoNT/F1 to N-glycan of SV2A, one could use our model to study contribution of each aromatic residue via production of single-point mutants of BoNT/F1 or to study impact of these aromatic residues at a larger scale by producing double- and triple-point mutants. These studies could be performed in vitro with surface plasmon resonance (combined with nanodiscs, as performed by Stern et al. [33]), pull-down assay, isothermal calorimetry or immunofluorescence, or in vivo by measuring LD50 (minimal dose to kill 50% of a population of mice) of BoNT/F1 mutants. Obviously, *in silico* approaches must be carefully validated experimentally [34]. Overall, the results previously acquired by our group in raft–protein interactions have shown excellent correlation between molecular modeling predictions and experimental verifications through a broad range of physicochemical, biochemical and cellular approaches [35,36,37,38].

Finally, we studied interaction of BoNT/F1 with gangliosides. Available crystal coordinates that describe neurotoxin binding of gangliosides unfortunately lack in ceramide content of gangliosides [8,39]. Indeed, the fact that during crystallization, sugar residues may freely diffuse in water without physical constraint of a membrane can lead to a biased interpretation of interaction along the +Z and −Z axes. In the crystal structure 3RSJ, ganglioside glycan interacts with GBS residues R1111, H1241 and W1250 [39], which were also identified in our simulations. In addition to these residues of the GBS, we identified several other ganglioside-binding residues that interact with the Hcc domain of BoNT/F1 in a region that is homologous to the synaptotagmin-binding pocket in BoNT/B [40,41]. All additional interactions allowed fine-tuning of binding of the Hcc domain of BoNT/F1 and thereby better stabilization of neurotoxin with the neural membrane surface.

In conclusion, our MDS provided relevant structural data for design of inhibitors directed against BoNT/F and for development of bioengineered BoNT/F as a strategy to enhance its therapeutic effect. The fact that BoNT/F shares the same major contact points as BoNT/A and BoNT/E to bind glycan content of SV2 makes it possible to design an inhibitor directed against BoNT/F, which could also be effective against BoNT/A and BoNT/E. This fact also opens a gate to design of a universal inhibitor directed against N-glycan binding of the three serotypes BoNT/A, BoNT/E and BoNT/F.

## Data Availability

Not applicable.

## Supplementary Materials

The following supporting information can be downloaded at: https://www.mdpi.com/article/10.3390/biom12121821/s1, Figure S1: Root Mean Square Deviation (RMSD) of BoNT/F1 and BoNT/A1 over time. Figure S2: RMSF of hSV2Ag in complex with BoNT/A1. Figure S3: RMSF of hSV2Ag in complex with BoNT/F1. Figure S4: Shield effect of Y1132 that disrupts the inter-molecular hydrogen bonds formed between BoNT/F1 and the backbone of hSV2Ag.
