# Supplementary material for: Structural Basis of Botulinum Toxin Type F Binding to Glycosylated Human SV2A: In Silico Studies at the Periphery of a Lipid Raft
Source: Biomolecules. 2022 Dec 6;12(12):1821. doi: 10.3390/biom12121821 (PMC9776016; doi:10.3390/biom12121821)

## Supplementary Materials

### Structural Basis of Botulinum Toxin Type F Binding to Glycosylated Human SV2A: In Silico Studies at the Periphery of a Lipid Raft

**Fodil Azzaz [1,*], Didier Hilaire [2] and Jacques Fantini [1]**

1 Fodil Azzaz, INSERM U_1072, Faculté de Médecine Nord, Bd Pierre Dramard, University of Aix-Marseille, 13015 Marseille, France
2 DGA (Direction Générale de L'armement)—DGA Maîtrise NRBC, 91710 Vert le Petit, France

* Correspondence: fodil.azzaz@etu.univ-amu.fr; Tel.: +33-651-942-957

**Content : Figures S1- S4**

**Figure S1: Root Mean Square Deviation (RMSD) of BoNT/F1 (blue curve) and BoNT/A1 (black curve) over time.**

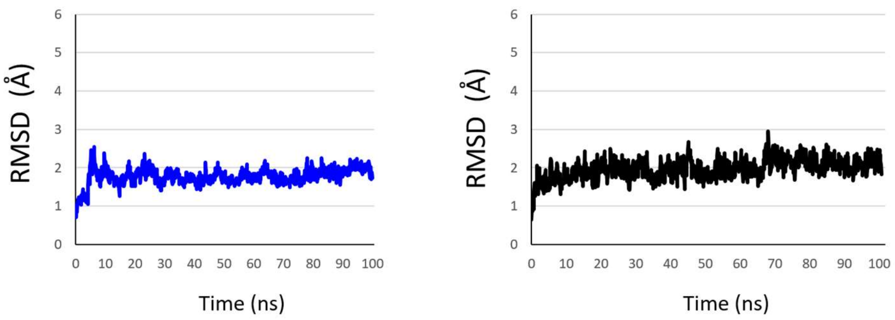

**Figure S2: RMSF of hSV2Ag in complex with BoNT/A1.** The RMSF corresponding to the transmembrane domains (TMD) are highlighted with blue brackets and the RMSF corresponding to the luminal domain (LD) is highlighted with a purple bracket **(A)**. RMSD of the whole structure of hSV2Ag over time **(B)**. RMSD of the TMD of hSV2Ag over time **(C)**. Cartoon representation of the structure of hSV2Ag (162-742) with its TMD colored in blue, LD colored in purple and the regions that present an RMSF above of 3 Å colored in black **(D)**.

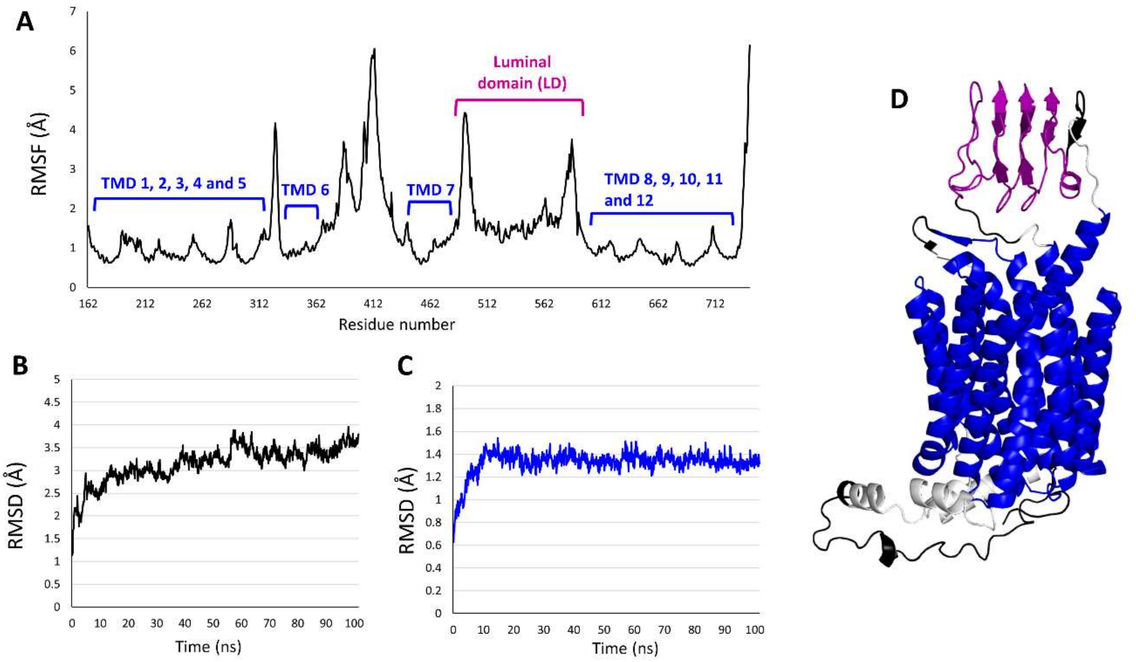

**Figure S3: RMSF of hSV2Ag in complex with BoNT/F1.** The RMSF corresponding to the transmembrane domains (TMD) are highlighted with blue brackets and the RMSF corresponding to the luminal domain (LD) is highlighted with a purple bracket **(A)**. RMSD of the whole structure of hSV2Ag over time **(B)**. RMSD of the TMD of hSV2Ag over time **(C)**. Cartoon representation of the structure of hSV2Ag (162-742) with its TMD colored in blue, LD colored in purple and the regions that present an RMSF above of 3 Å colored in black **(D)**.

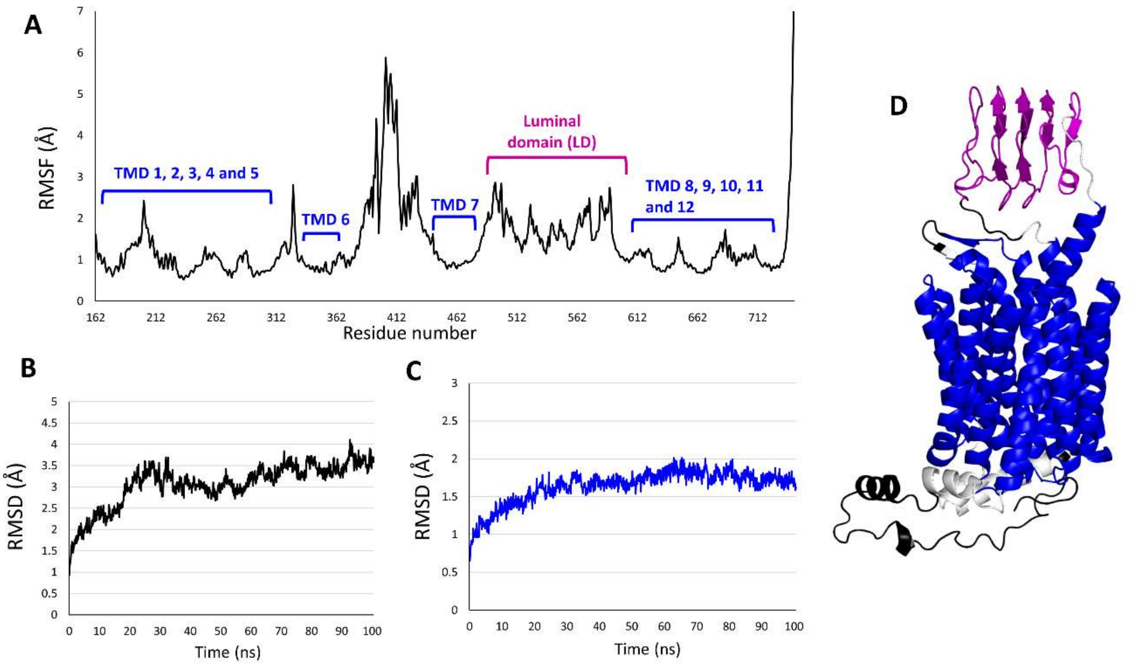


**Figure S4: Shield effect of Y1132 that disrupts the intermolecular hydrogen bonds formed between BoNT/F1 and the backbone of hSV2Ag.** Molecular details of the evolution of BoNT/F1-SV2A complex at 23 ns **(A)** 35 ns **(B)** and 100 ns **(C)**. BoNT/F1 residues are depicted as sticks colored by atom name and the SV2A backbone is depicted as spheres colored by atom name. The intermolecular hydrogen bonds are indicated with black asterisks and the intramolecular hydrogen bonds with red asterisks.

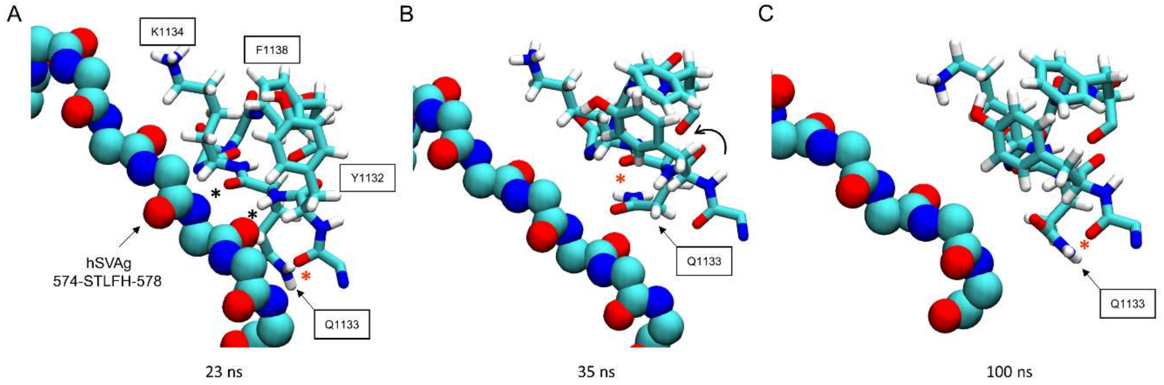

Supplement: Supplementary file 1 [file biomolecules-12-01821-s001.zip › biomolecules-2034678-supplementary.pdf]
